# Prognostic model on overall survival in elderly nasopharyngeal carcinoma patients: a recursive partitioning analysis identifying pre-treatment risk stratification

**DOI:** 10.1186/s13014-023-02272-x

**Published:** 2023-06-23

**Authors:** Ying Li, Youliang Weng, Zongwei Huang, Yuhui Pan, Sunqin Cai, Qin Ding, Zijie Wu, Xin Chen, Jun Lu, Dan Hu, Sufang Qiu

**Affiliations:** 1 Clinical Oncology School of Fujian Medical University, Fujian Cancer Hospital, Fujian, China; 2 Fujian Key Laboratory of Translational Cancer Medicine, Fujian, China; 3 Fujian Provincial Key Laboratory of Tumor Biotherapy, Fujian, China

**Keywords:** Nasopharyngeal carcinoma, Elderly patients, Intensity-modulated radiotherapy, Recursive partitioning analysis, Prognostic model

## Abstract

**Background:**

We aimed to evaluate the optimal management for elderly patients with nasopharyngeal carcinoma (NPC) with intensity-modulated radiotherapy (IMRT).

**Methods:**

A total of 283 elderly patients with NPC diagnosed from 2015 to 2019 were enrolled in the study. Overall survival (OS) was the primary endpoint. Univariate and multivariate Cox regression analyses were preformed to identify potential prognostic factors. The recursive partitioning analysis (RPA) was used for risk stratification. Kaplan-Meier survival curves were applied to evaluate the survival endpoints, and log-rank test was utilized to assess differences between groups. The prognostic index (PI) was constructed to further predict patients’ prognosis displayed by nomogram model. The area under the receiver operating characteristic (ROC) curves (AUC) and the calibration curves were applied to assess the effectiveness of the model.

**Results:**

Based on RPA-based risk stratification, we demonstrated that elderly NPC patients who were treated with IC followed by RT had similar OS as those with induction chemotherapy (IC) combined with concurrent chemoradiotherapy (CCRT) in the middle- (stage I-III and pre-treatment EBV > 1840 copies/ml) and high-risk groups (stage IVA). IMRT alone may be the optimal treatment option for the low-risk group (stage I-III with pre-treatment EBV ≤ 1840 copies/ml). We established an integrated PI which was indicted with stronger prognostic power than each of the factors alone for elderly NPC patients (The AUC of PI was 0.75, 0.80, and 0.82 for 1-, 3-, 5-year prediction of OS, respectively).

**Conclusion:**

We present a robust model for clinical stratification which could guide individual therapy for elderly NPC patients.

**Supplementary Information:**

The online version contains supplementary material available at 10.1186/s13014-023-02272-x.

## Introduction

Nasopharyngeal carcinoma (NPC), a highly aggressive malignancy, is associated with unique epidemiological and geographical attributes, primarily prevalent in Southern China [1]. The age distribution peaks between 45–59 years old in epidemic regions [2]. According to the statistics, 60% of new cancer cases occur in individuals aged over 65 [3]. As the global population ages, the proportion of elderly individuals with cancer is progressively increasing, elderly NPC cases are not rare [4]. However, the optimal management for this demographic remains unclear.

Radiotherapy (RT) is the cornerstone for NPC treatment. Precise target volume coverage and dosimetric advantages make intensity-modulated radiotherapy (IMRT) the preferred option [5]. Studies have demonstrated a significant improvement in 5-year survival rates and reduced complications when combining RT with chemotherapy [6]. Presently, induction chemotherapy (IC) combined with concurrent chemoradiotherapy (CCRT) has been the favored treatment modality for locoregionally advanced NPC patients [5]. However, in elderly individuals, poor performance status, coexisting ailments, and reduced organ function are common, leading to increased health burdens [7]. The representation of elderly patients in clinical trials has typically been limited or excluded, rendering the efficacy of standard active treatments in this vulnerable population inconclusive [8, 9]. Poorer survival has been observed in elderly NPC patients compared to the younger counterparts, possibly due to increased sensitivity to RT and chemotherapy and reduced tolerance to treatment toxicity [10–12]. Hence, personalized management with appropriate risk stratification is vital to avoid under and overtreatment in elderly individuals.

Elderly individuals with cancer often present with comorbidities, such as infection, inflammation, and organ dysfunction, resulting in a complex decision-making process for treatment [8]. Notably, the level of comorbidity has been established to impact the prognosis of elderly patients with NPC [13]. Chronic inflammation is a hallmark of cancer progression and a key process of aging [14]. Inflammatory markers are suggested as predictors of unfavorable health outcomes, particularly among older individuals with NPC [15]. Furthermore, Epstein-barr virus (EBV) DNA level, functional status, nutrition status, and other factors, such as telomere length and DNA methylation, have been also examined for their prognostic value [16]. Each of these factors may adversely affect treatment tolerance and prognosis in elderly patients. Therefore, identifying robust prognostic factors by combining elderly patient characteristics could facilitate the development of more accurate models for risk stratification to assist patients with varying risk profiles in making appropriate decisions regarding treatment selection, which would be of great significance to the elderly with NPC.

In this study, we focused on the survival and prognosis in elderly NPC patients in the IMRT era. The identification of prognostic factors and establishment of a risk stratification model via recursive partitioning analysis (RPA) were undertaken to predict overall survival (OS), providing guidance to clinicians in selecting the optimal treatment options for senior NPC patients.

## Methods and materials

### Patients

A retrospective analysis was conducted on a cohort of 354 elderly NPC patients who were first diagnosed at Fujian Cancer Hospital, China, between 2015 and 2019. The inclusion criteria included: (1) pathologically confirmed NPC; (2) age ≥ 65 years; (3) Karnofsky performance score (KPS) ≥ 70; (4) receipt of IMRT; (5) availability of pre-treatment EBV DNA levels. The exclusion criteria were as follows: (1) patients with distant metastasis when diagnosed; (2) prior anti-tumor treatment; (3) palliative treatment; (4) treatment abandonment; (5) severe comorbidities; (6) loss to follow-up. The patient selection process is outlined in Supplementary Fig. S1. The Ethics Committee of Fujian Cancer Hospital has reviewed and approved the study (K2022-203-01).

### Evaluation and treatment

Patients underwent pretreatment evaluations comprising medical history, physical examination, hematological testing, nasopharyngoscopy with biopsy, magnetic resonance imaging (MRI) of the head and neck region, computed tomography (CT) of the chest, abdominal ultrasonography, and whole-body bone emission computed tomography (ECT). Plasma EBV DNA levels were quantified by real-time quantitative polymerase chain reaction (qPCR) prior to treatment, as previously detailed elsewhere [17]. Imaging-based restaging according to the 8th AJCC staging system was performed by two radiologists.

Radical IMRT was administered using simultaneous integrated boost (SIB) with 6 MV photons. All patients utilized a thermoplastic mask for immobilizing with supine position. Target volume delineation and organs at risk (OARs) identification were assisted by the fusion of CT/MRI images. Gross tumor volume (GTV) encompassed all visible disease, including nasopharyngeal primary tumors (GTV-T) and positive lymph nodes (GTV-N) identified by imaging, clinical examination, and endoscopic findings. Clinical target volume (CTV) was defined as GTV and its surrounding subclinical lesions. Planning target volume (PTV) was established as a safety boundary around GTV/CTV with an additional 3 mm margin to account for positional errors and intrinsic organ movements. Radiation doses prescribed for NPC were delineated according to our institution’s guidelines, with a prescribed radiation dose of 60–76 Gy for PTV-GTV-T, 60–74 Gy for PTV-GTV-N, and 50–62 Gy for PTV-CTV in 30 to 38 fractions, given once per day, five times per week. Dose constraints for OARs were determined in accordance with the RTOG 0225 protocol [18].

Patients were given 1–6 cycles of IC, which was largely determined by the physician based on the patient’s pretreatment features or the tolerance of chemotherapy. Platinum-based IC regimens were commonly combined with gemcitabine, paclitaxel, docetaxel + 5-fuorouracil or other regimens every 3 weeks. 144 patients received 1–3 cycles of platinum-based concurrent chemotherapy. The application of adjuvant chemotherapy and targeted therapy was on the basis of the physician’s judgment and tumor’s status.

### Geriatric assessment

The age-adjusted Charlson comorbidity index (ACCI) was utilized as an evaluation tool for comorbid conditions across various organ systems at the time of diagnosis. It regards age of olders as correction variable and has been found to be an independent prognostic indicator for long-term survival [19]. Details on scoring methodology for ACCI were mentioned else [20]. In addition, the neutrophil-lymphocyte ratio (NLR) was employed as an inflammatory index, calculated as the ratio of absolute neutrophils count to absolute lymphocytes count at admission [21]. The albumin-bilirubin (ALBI) score, which reflects the general status and nutritional condition, was calculated using the formula: {log10 [total bilirubin (umol/L)] × 0.66} + [albumin (g/L) × 0.085] [22].

### Follow-up and outcome

Upon completing treatment, all patients underwent follow-up assessments every 3 months during the first 2 years, every 6 months in the following 3 years, and annually thereafter. Loss to follow-up referred to the frequency with which patients have no follow-up data after therapy at all. Survival and tumor status were documented using clinical records or telephone communication, with the final follow-up time on March 31, 2022. Recurrence or metastasis was confirmed by pathology whenever possible. If unavailable, it was diagnosed by at least 2 imaging findings.

The primary outcome of this study was OS, defined as the interval from IMRT completion to death or the last follow-up. Progression-free survival (PFS) was defined as the interval from IMRT completion to the onset of local or regional recurrence, distant metastasis, death, or the last follow-up. Locoregional relapse-free survival (LRFS) and distant metastasis-free survival (DMFS) were defined as the interval from IMRT completion to death and the diagnosis of locoregional progression or distant metastasis, respectively. Patients underwent salvage therapy according to their wishes and physicians’ decisions if tumor progression occurred.

### Construction of the prognostic model

Univariate and multivariate Cox regression analyses were conducted to identify potential prognostic factors. Variables that demonstrated observably significance in the multivariate analysis were included in a risk stratification model for OS using RPA. The RPA was used to select the best node in each split to get the best patient groups in each step, based on classification and regression tree (CART) analysis [23]. Each subgroup would be subsequently split into smaller groups until a specified stopping criterion is reached or further divisions could no longer be made. A prognostic index (PI) was established using clinical parameters by a stepwise process of backward selection, where insignificant parameters were removed using Akaike information criterion (AIC) for model fitting [24]. The final PI was selected based on the smallest AIC. A nomogram was presented using the “rms” R package to visualize the score of each parameter on the point scale. The area under the receiver operating characteristic (ROC) curves (AUC) and the calibration curves were depicted to validate the prediction accuracy of the PI for 1/3/5-year OS. The calibration curves close to ideal line was considered as the best prediction, and an AUC value more than 0.7 was seen to be significant predictive performance.

### Statistics

Survival outcomes were assessed using Kaplan-Meier survival analysis, and differences between groups were compared using the log-rank test. Pairwise comparisons between groups were carried out using the Bonferroni test. R software (https://www.r-project.org, v3.6.2) and SPSS Statistics v25.0 were conducted for all statistical analysis. A *P*-value < 0.05 was deemed statistically significant, unless otherwise indicated.

## Results

### Patient characteristics and survival

Totally 354 elderly patients were diagnosed with NPC in our center from January 2015 to December 2019. Among them, 7 cases received previous anti-tumor treatment, 12 cases received palliative treatment, 28 cases abandoned or interrupted treatment. thus excluding them from this study. Then 307 patients completed radical treatment based on IMRT in our center. After further exclusion of 6 patients who were lost to follow-up, 16 with confirmed distant metastasis, and 2 with controversial metastasis, we were left with a cohort of 283 patients. These patients were between the ages of 65 and 87, and included 203 males and 80 females. The median NLR was 2.16 (range 0.61–7.84). The range of ALBI for the whole cohort was − 3.62 to -1.60 (median = −2.70). The median pre-treatment plasma EBV DNA level was 2310 copies/mL with a range of 0-9.85×10^5^ copies/mL. Treatment regimens for the cohort were as follows: 57 (20.14%) patients were treated with RT alone, 82 (28.98%) patients received IC followed by RT, 29 (10.25%) patients received CCRT, and 115 (40.64%) patients received IC combined with CCRT. Table 1 showed the baseline characteristics of the cohort.

**Table 1:** Baseline characteristics of patients

Characteristic	No. of patients (%)
Age (years)	65–87 (Median = 67)
Gender	
Male	203 (71.73)
Female	80 (28.27)
KPS	
90	225 (79.51)
< 90	58 (20.49)
ALBI	-3.62 to -1.60 (Median = -2.70)
NLR	0.61–7.84 (Median = 2.16)
ACCI	
2	121 (42.76)
3	93 (32.86)
4	41 (14.49)
≥ 5	28 (9.89)
T classification	
T1	37 (13.07)
T2	51 (18.02)
T3	106 (37.46)
T4	89 (31.45)
N classification	
N0	30 (10.60)
N1	127 (44.88)
N2	101 (35.69)
N3	25 (8.83)
Stage	
Stage I	6 (2.12)
Stage II	41 (14.49)
Stage III	128 (45.23)
Stage IVA	108 (38.16)
EBV DNA_pre_ (copies/mL)	0-9.85×10^5^ (Median = 2310)
Treatment modality	
RT alone	57 (20.14)
IC + RT	82 (28.98)
CCRT	29 (10.25)
IC + CCRT	115 (40.64)

ACCI, age-adjusted Charlson comorbidity index; ALBI, albumin-bilirubin grade; CCRT, concurrent chemoradiothrapy; EBV, Epstein-barr virus; EBV DNA_pre_, pre-treatment EBV DNA level; IC, induction chemotherapy; KPS, Karnofsky performance score; NLR, neutrophil-to-lymphocyte ratio; RT, radiotherapy

Median follow-up time was 35.7 months (range 2–84 months). In total, 54 (19.08%) patients died, 18 (6.36%) patients experienced local or regional recurrence, 19 (6.71%) developed distant failure, 70 (24.73%) suffered disease progression at their last follow-up. Estimated 3-year OS, PFS, LRFS and DMFS rates were 82.35%, 76.47%, 79.61% and 80.58%, respectively (Supplementary Fig. S2).

### Identification of prognostic factors

**Fig. 1:**
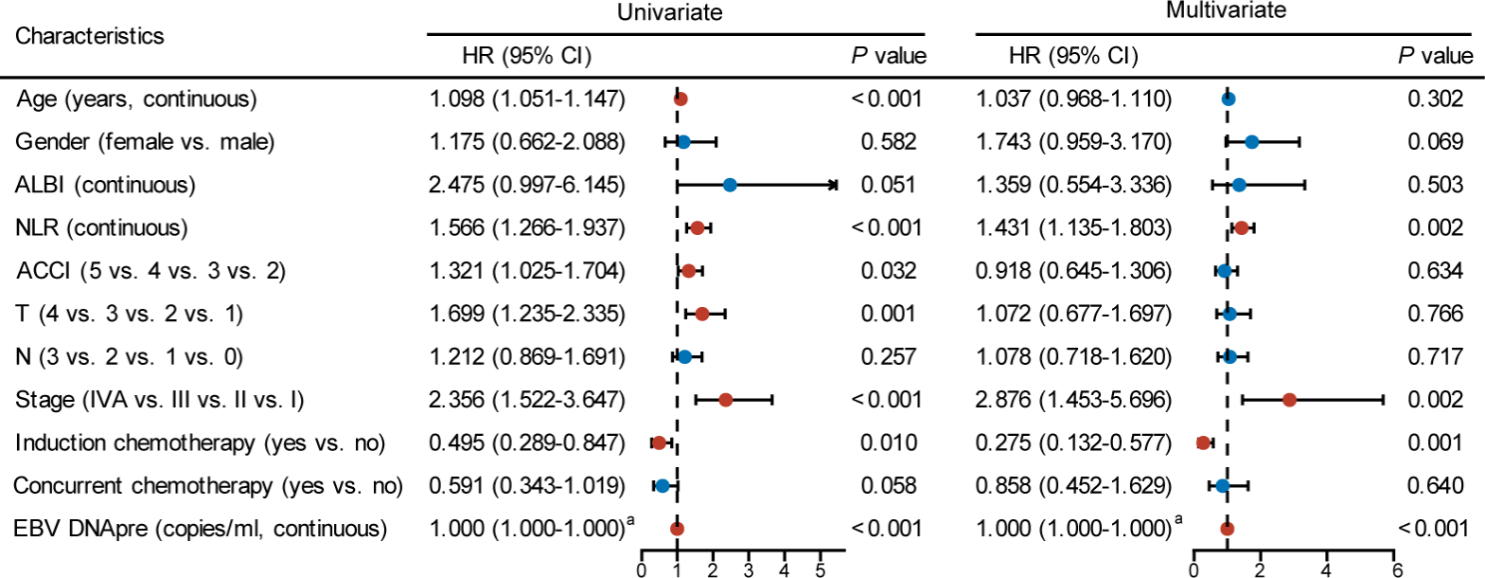
Univariate and multivariate analysis of prognostic factors for overall survival. HR, hazard ratio; CI, confidence interval; ALBI, albumin-bilirubin grade; NLR, neutrophil-to-lymphocyte ratio; ACCI, age-adjusted Charlson comorbidity index; EBV, Epstein-barr virus; EBV DNA_pre_, pre-treatment EBV DNA level. ^a^ The HR was > 1.000. The red dot represents the significant prognostic factor (*P* < 0.05); Blue dot represents non-significant factor

We evaluated several parameters to identify potential prognostic factors. Among these variables, it revealed that age (HR: 1.098, 95%CI: 1.051–1.147, *P* < 0.001), NLR (HR: 1.566, 95%CI: 1.266–1.937, *P* < 0.001), ACCI (HR: 1.321, 95%CI: 1.025–1.704, *P* = 0.032), T classification (HR: 1.699, 95%CI: 1.235–2.335, *P* = 0.001), clinical stage (HR: 1.356, 95%CI: 1.522–3.647, *P* < 0.001), IC (HR: 0.495, 95%CI: 0.289–0.847, *P* = 0.010), and pre-treatment plasma EBV DNA levels (HR: 1.000, 95%CI: 1.000–1.000, *P* < 0.001) significantly affected OS in univariate analysis (Fig. 1). Multivariate analysis further indicated that a higher NLR (HR: 1.431, 95%CI: 1.135–1.803, *P* = 0.002), stage IVA (HR: 2.876, 95%CI: 1.453–5.696, *P* = 0.002), and higher pre-treatment plasma EBV DNA levels (HR: 1.000, 95%CI: 1.000–1.000, *P* < 0.001) were poorer independent risk factors for OS. Conversely, IC was an independent protective factor for OS (HR: 0.275, 95%CI: 0.132–0.577, *P* = 0.001) .

### RPA risk stratification and subgroup analysis

**Fig. 2:**
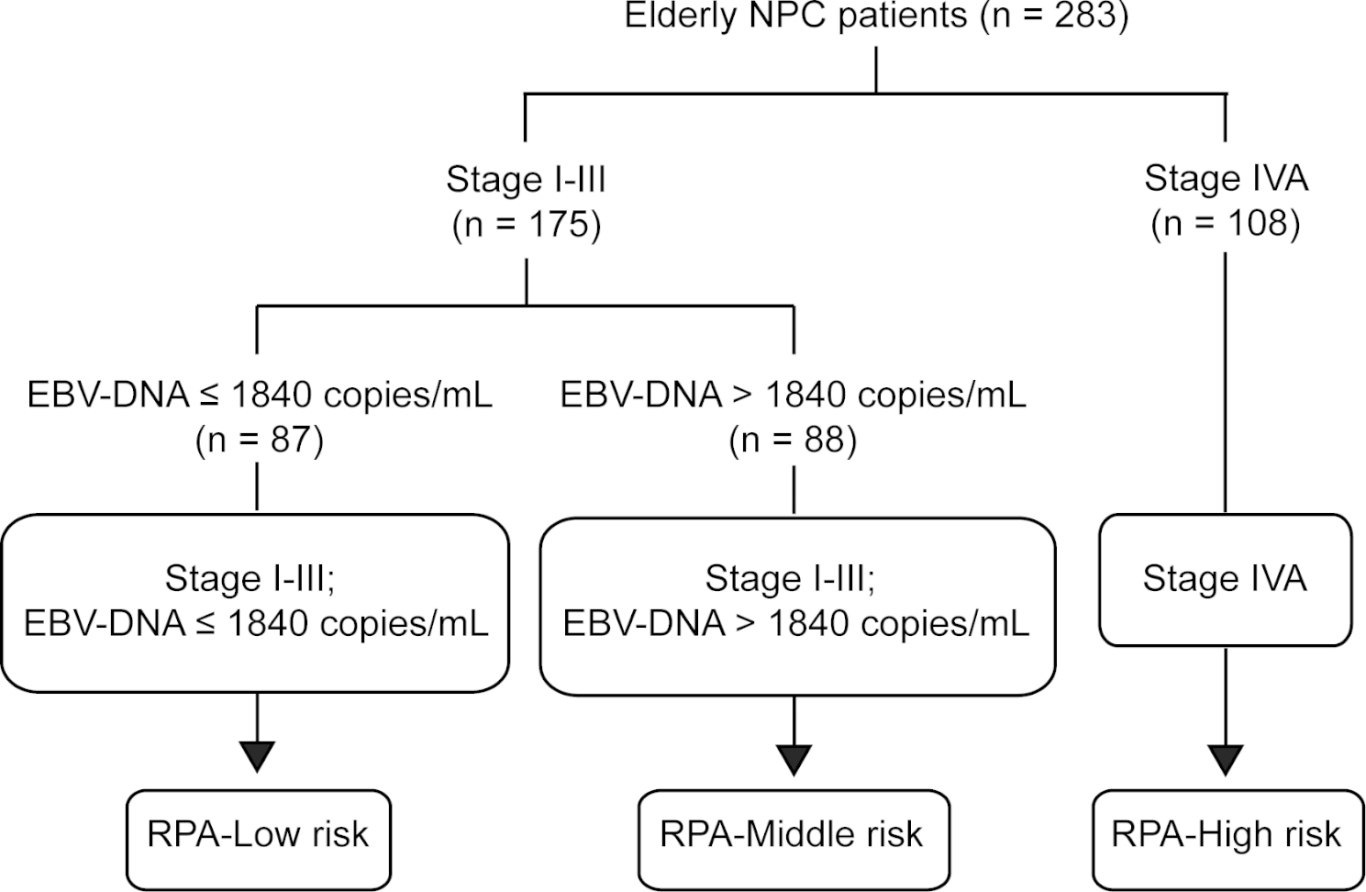
Prognostic stratification by recursive partitioning analysis in elderly NPC for predicting overall survival. NPC, nasopharyngeal carcinoma; EBV, Epstein-barr virus; EBV DNA_pre_, pre-treatment EBV DNA level; RPA, recursive partitioning analysis

**Fig. 3:**
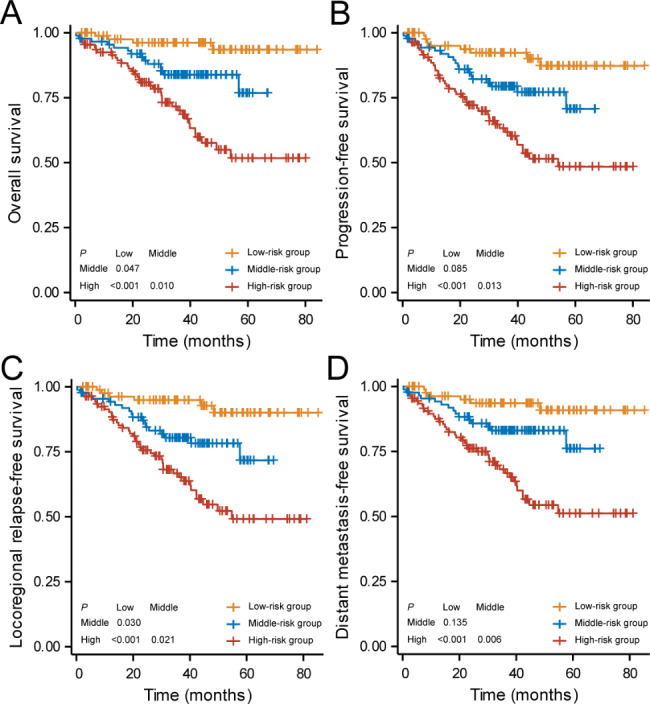
Kaplan-Meier survival curves of (**A**) overall survival, (**B**) progression-free survival, (**C**) relapse-free survival and (**D**) distant metastasis-free survival in stratified risk groups by recursive partition analysis. RPA, recursive partitioning analysis

We selected 3 pre-treatment independent prognostic predictors including NLR, clinical stage, and pre-treatment plasma EBV DNA levels for risk stratification based on OS. Finally, we established a risk model by the RPA algorithm which consisted of 3 groups, with 87 (30.74%), 88 (31.10%), and 108 (38.16%) patients assigned to low-, middle- and high-risk groups, respectively (Fig. 2). The low-risk group refers to patients in stage I-III with pre-treatment EBV DNA levels ≤ 1840 copies/mL. The middle-risk group refers to patients in stage I-III with pre-treatment EBV DNA levels > 1840 copies/mL. The high-risk group refers to patients in stage IVA. The RPA-based risk model showed satisfactory prognostic value for OS of elderly NPC patients as shown in Fig. 3A (high-risk group vs. low-risk group: HR: 8.847, 95%CI: 4.760-16.442, *P* < 0.001; high-risk group vs. middle-risk group: HR: 2.442, 95%CI: 1.402–4.251, *P* = 0.010; middle-risk group vs. low-risk group: HR: 3.577, 95%CI: 1.420–9.012, *P* = 0.047). There was also a significant difference in LRFS among the three risk groups (high-risk group vs. low-risk group: HR: 6.609, 95%CI: 3.708–11.781, *P* < 0.001; high-risk group vs. middle-risk group: HR: 3.139, 95%CI: 1.410–6.989, *P* = 0.030; middle-risk group vs. low-risk group: HR: 2.112, 95%CI: 1.262–3.535, *P* = 0.021; Fig. 3C). In addition, patients in the high-risk group had poorer PFS (high-risk group vs. low-risk group: HR: 5.195, 95%CI: 3.000-8.995, *P* < 0.001; high-risk group vs. middle-risk group: HR: 2.158, 95%CI: 1.311–3.550, *P* = 0.013, Fig. 3B) and DMFS (high-risk group vs. low-risk group: HR: 6.304, 95%CI: 3.514–11.309, *P* < 0.001; high-risk group vs. middle-risk group: HR: 2.479, 95%CI: 1.454–4.227, *P* = 0.006, Fig. 3D) than those in the low- and middle-risk groups.

**Fig. 4:**
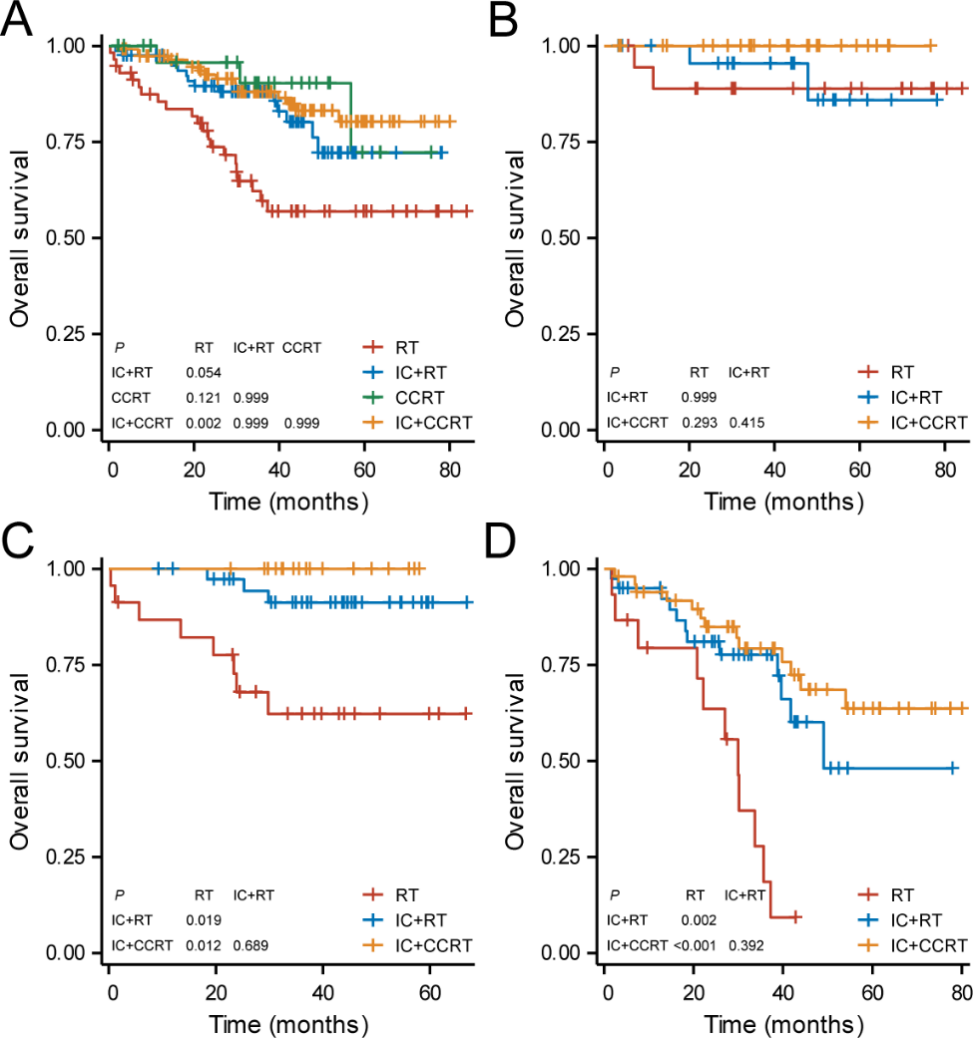
Kaplan-Meier survival curves of overall survival between patients with different treatment strategies in (**A**) the whole cohort, (**B**) low-risk group, (**C**) middle-risk group, and (**D**) high-risk group. RT, radiotherapy; IC, induction chemotherapy; CCRT, concurrent chemoradiothrapy

We investigated the survival benefits of different treatment strategies for elderly NPC patients. It suggested that patients who received with IC + CCRT achieved higher OS as compared to those treated with RT alone (IC + CCRT vs. RT, HR: 3.143, 95%CI: 1.550–6.373, *P* = 0.002). However, the survival benefits of IC + RT and CCRT were not noted than RT alone (IC + RT vs. RT, HR: 2.393, 95%CI: 1.211–4.726, *P* = 0.054; CCRT vs. RT, HR: 3.787, 95%CI: 1.638–8.757, *P* = 0.121; Fig. 4A). No significant differences were found between IC + RT, CCRT and IC + CCRT group, with *P* > 0.05 for each of the two groups. Further analysis was performed to explore the survival benefits of treatment within three subgroups based on RPA risk model. Given the limited number of patients treated with CCRT, they weren’t included in further analysis. In the low-risk group, no significant difference in OS was found between patients with RT, IC + RT or IC + CCRT (*Ps* > 0.05, Fig. 4B), indicating that elderly NPC patients in this subgroup could achieve sufficient survival benefits from RT alone. In the middle-risk group, patients treated with IC + RT and IC + CCRT achieved higher OS compared to those with RT alone, which was similar to the result observed in the high-risk group (*Ps* < 0.05). However, no significant difference was found between patients treated with IC + RT and IC + CCRT in these two groups (*Ps* > 0.05, Figs. 4C and 4D).

### Construction of nomogram model for OS in elderly NPC patients

**Fig. 5:**
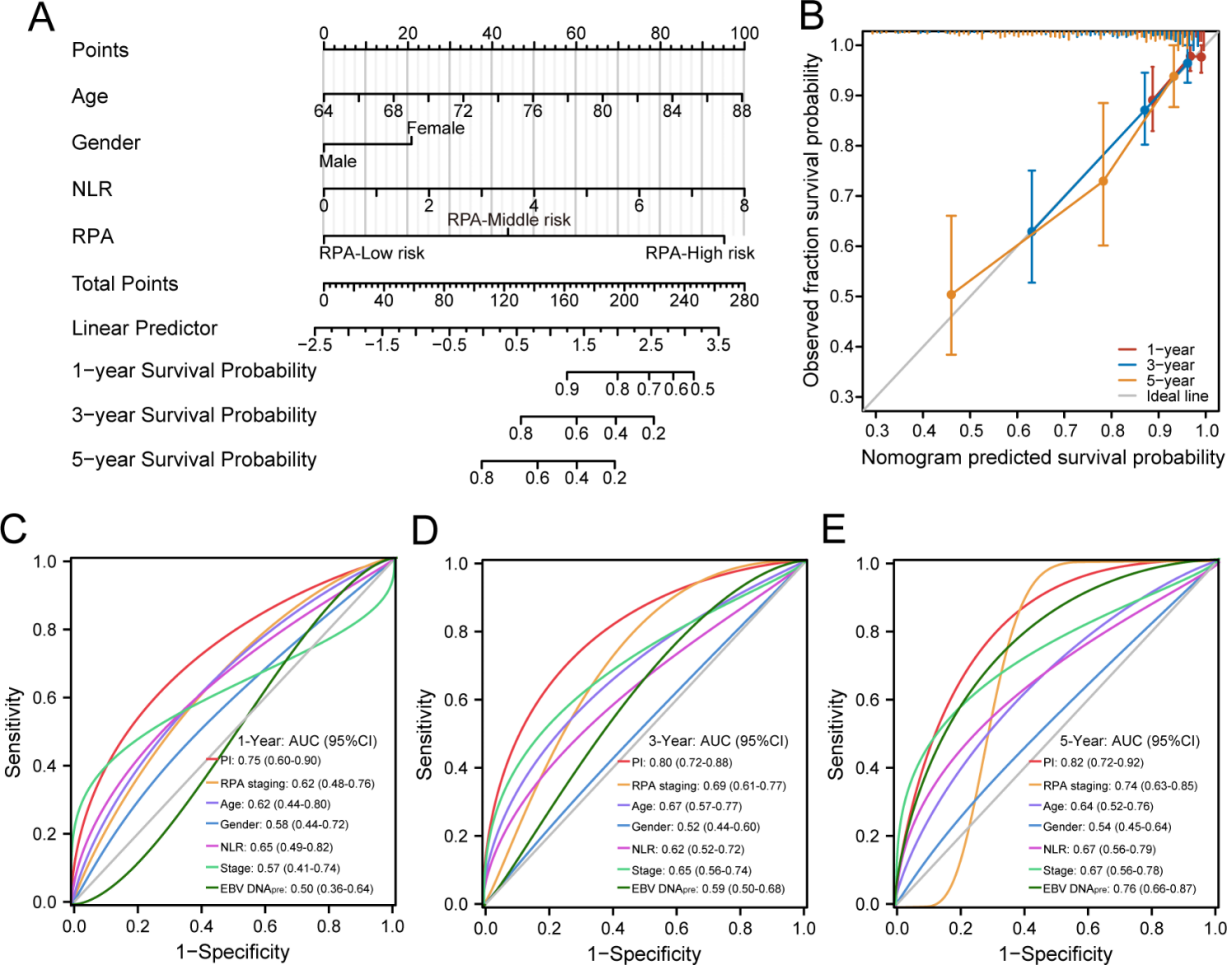
Construction and validation of the predictive model in elderly NPC patients. (**A**) Nomogram model established by prognostic index to estimate the 1-, 3- and 5-year survival possibility. (**B**) The calibration curves for validation of the nomogram. (**C-E**) The ROC curves comparing the accuracy of PI, RPA grouping and the other prognosis factors for predicting 1-, 3- and 5-year survival rate. NLR, neutrophil-to-lymphocyte ratio; RPA, recursive partitioning analysis; AUC, the area under the receiver operating characteristic curves; CI, confidence interval; PI, prognostic index; EBV, Epstein-barr virus; EBV DNA_pre_, pre-treatment EBV DNA level. TPR, true positive rate; FPR, false positive rate

To further predict patient outcomes, we enrolled all relevant prognosis factors, including age, gender, NLR, ACCI, ALBI, T stage, N stage and RPA grouping to construct a prognostic model. Finally, the PI was developed utilizing age, gender, NLR and RPA grouping for robust clinical stratification. A nomogram showed the individual assessment based on the PI that is tailored to each elderly NPC patient (Fig. 5A). The PI score was calculated based on the score of each prognostic variable, which also enabled estimation of individual probabilities for 1-, 3-, and 5-year OS. The calibration curves showed the good performance of the nomogram (Fig. 5B). Additionally, a comparative analysis was performed on the prognostic performance of the PI in comparison to other factors, which showed that when combined with RPA grouping, our PI presented a superior prognostic discriminatory power when compared to other individual indicators in terms of predicting the OS of elderly NPC. The AUC of PI was determined to be 0.75, 0.80, and 0.82 for 1-, 3-, and 5‐year OS prediction, respectively, thus significantly enhancing the predictive performance when compared to RPA grouping, age, gender, NLR, stage, and pre-treatment EBV DNA levels (Figs. 5C-5E).

## Discussion

In current study, we developed an RPA-based risk stratification which integrated clinical stage and pre-treatment EBV DNA level for elderly NPC patients who underwent IMRT. It was shown that patients in the high-risk group exhibit inferior OS, PFS, LRFS and DMFS compared to those in the low- and middle-risk groups. Our findings revealed that patients in the low-risk group do not benefit from additional IC or concurrent chemotherapy compared to RT alone, whereas IC followed by RT or IC combined with CCRT may be the optimal treatment opinions for patients in the middle- and high-risk groups. In addition, we established an integrated RPA-based PI for elderly NPC, which exhibits superior prognostic performance compared to other single factor.

Given the aging population in China, the burden of NPC is growing [25]. Some characteristics of NPC display regional variations [2]. Over he age of 65 represents the second peak of the increasing incidence of NPC in low-incidence regions [26]. Epidemiological studies have revealed that the proportion of elderly NPC patients (≥ 60 years old) is 35.0% in non-endemic areas, which is higher than that in endemic areas (13.8%) [27, 28]. Management of IMRT for elderly NPC patients remains challenges. Some retrospective studies have suggested that RT combined with chemotherapy could improve survival rates in elderly NPC patients receiving conventional RT [27]. Conversely, others have argued that chemotherapy may not provide additional more survival benefits and may even bring more therapeutic toxicity in elderly NPC patients in the IMRT era [29]. In a comparison between CCRT and RT alone, grade 3–4 severe mucositis and dermatitis were observed more frequently in the former, and comorbidities were found to increase the likelihood of severe toxic reactions [4]. A study conducted by Wen *et al.* also demonstrated that CCRT was associated with higher rates of hematological adverse reactions, such as leukopenia, neutropenia, and thrombocytopenia [30]. Additionally, Ou *et al.* observed that patients receiving IC combined with CCRT had lower rates of grade 3–4 late toxicities compared to those receiving CCRT alone in non-endemic areas [31]. However, in an analysis of NPC patients over 60 years old, the addition of IC did not significantly affect survival CCRT, but instead increased grade 3 to 4 acute toxicities [9]. The differences in the pharmacokinetics and pharmacodynamics of chemotherapeutic agents in patients of different ages, with the same drugs tending to have a higher toxicity profile in the elderly [32]. Thus, reducing treatment-related toxicity is a focus of concern in treatment decisions [33, 34]. However, we lacked the data on treatment-related toxicities in this study. In terms of efficacy, our study showed that the survival benefits of IC prior to RT was comparable to that of IC combined with CCRT as a first-line treatment in selected middle- and high-risk patients. A less intensive treatment regimen was warranted for low-risk patients. However, we didn’t find the benefits of CCRT in elderly patients with NPC, which is consistent with previous reports [35]. Furthermore, multivariate analyses indicated that IC was an independent protective factor for OS, while concurrent chemotherapy was not. This outcome may be attributed to the greatly enhanced curative effect of IMRT on NPC [36]. In addition, elderly patients may have lower tolerance owing to multiple comorbidities, poor organ functional status and performance status [11]. Thus, the magnitude of the impact of concurrent chemotherapy may be limited.

Up to now, only a limited number of studies have evaluated clinical endpoints in elderly NPC patients, and none have taken into account risk stratification [9, 12, 35]. The RPA model has been widely used in many malignancies for prognosis stratification according to homogeneous survival performance [37]. Notably, we considered both anatomical prognostic factors (i.e. AJCC stage) and some non-anatomic one, such as plasma EBV DNA level and parameters about geriatric assessment, which were closely associated with survival outcomes of elderly NPC patients. Therefore, taking prognostic factors before treatment into consideration, our RPA model considered clinical stage as the first split and pre-treatment EBV DNA level as the second split. It has been demonstrated the value of EBV DNA in NPC prognostication and as an important complement to the AJCC staging, which contributing to stratify patients with risk subgroups [38–40]. However, the optimal cut-off value of pre-treatment EBV DNA remains controversial. Some studies suggest that levels above 4000 copies/mL or 8000 copies/mL are predictive of poor prognosis, others report levels below 1500 copies/mL as prognostically significant in elderly NPC patients [41–43]. In our study, we found that a pre-treatment EBV DNA cut-off value of 1840 copies/mL was a good predictor of prognostic stratification for elderly NPC patients with clinical stage I to III, close to the results of Guo *et al.*, who obtained 2000 copies/mL of EBV DNA level before treatment by using RPA analysis [38].

In terms of heterogeneity in this population, the assessment of prognosis remains challenging in elderly NPC patients. Age is known to affect various aspects of tumor, including its growth mode, genomic stability, protein homeostasis, tissue repair ability, metabolism, intercellular communication, *etc.* [44]. Prior research has shown that old age is an adverse factor for survival and an independent risk factor for lethal nasopharyngeal necrosis after NPC re-irradiation [45, 46]. However, our study findings suggest that age may not be a strong prognostic factor in the elderly population, which is consistent with the findings of Chan *et al.* and Li *et al.* [47, 48]. Age alone should not be the sole basis for treatment decisions in elderly NPC patients, as other factors such as functional status, comorbidity, nutrition, and chronic inflammation may also impact prognosis [49]. Comorbidity is an important prognostic factor for elderly patients, and it may contribute to patient weakness, delay in treatment completion, and worsening of treatment-related toxicity [13]. The ACCI has been shown to predict survival and influence clinical manifestation, therapeutic interventions, and outcomes of NPC patients after RT [19]. The prognostic value of inflammatory markers have also been identified in various cancers [21]. A high NLR has been found to be associated with frailty in the elderly with cancer, indicating a decrease in physiologic reserve and an increase in vulnerability to multiple organ systems, which leads to poor health outcomes [50]. Although the predictive model based on clinical parameters has been shown to have favorable discriminative performance, it is suggested that more biomarkers should be taken into account in elderly patients. Future studies should consider the comprehensive and professional geriatric assessment, which could better strengthen the management of elderly patients and inform clinical decision making for this patient population. Our results demonstrated that the newly integrated PI, which combined the RPA model and additional biomarkers, had greater predictive power than using each biomarker separately. These findings suggest that the PI could serve as a satisfactory predictive indicator in elderly NPC patients.

In this study, we construct a risk stratification model to explore the optimal treatment strategy for elderly NPC patients. The inclusion of a large number of recently treated patients within a relatively short timeframe may be one of the main strengths of our study, which can provide valuable insights into current treatment practices and outcomes in clinical practice. Nonetheless, we acknowledge that there are several limitations to our study. Firstly, it is a retrospective analysis, we only chose OS as the primary clinical outcome to avoid the possible bias from changes in the follow-up programme. Secondly, comprehensive geriatric assessments by geriatricians were not available for our patients, but we attempted to consider several aspects that could impact the elderly. Thirdly, we recognize that biases related to heterogeneity in chemotherapy prescription, including the diversity of treatment regimens and chemotherapy cycles, are likely to persist. Furthermore, due to the limited number of cases with CCRT alone, we didn’t analyze these patients separately, and a larger sample size is required to explore the long-term outcomes of this patient population. Finally, limited to the retrospective nature, this study lacked information on treatment-related adverse effects for different treatment methods.

## Conclusions

In conclusion, we developed an RPA model for risk stratification and our constructed PI is robust in predicting survival in elderly patients with NPC which could serve as a tool in treatment decision-making for physicians.

## Electronic Supplementary Material

Below is the link to the electronic supplementary material

Supplementary Material 1

## Data Availability

The datasets generated during and/or analysed during the current study are available from the corresponding author on reasonable request.

## Abbreviations

NPC: Nasopharyngeal carcinoma
RT: Radiotherapy
IMRT: Intensity-modulated radiotherapy
IC: Induction chemotherapy
CCRT: Concurrent chemoradiotherapy
EBV: Epstein-barr virus
RPA: Recursive partitioning analysis
PI: Prognostic index
OS: Overall survival
KPS: Karnofsky performance score
MRI: Magnetic resonance imaging
CT: Computed tomography
ECT: Emission computed tomography
qPCR: Quantitative polymerase chain reaction
SIB: Simultaneous integrated boost
OARs: Organs at risk
GTV: Gross tumor volume
CTV: Clinical target volume
PTV: Planning target volume
ACCI: Age-adjusted Charlson comorbidity index
NLR: Neutrophil-lymphocyte ratio
ALBI: Albumin-bilirubin
PFS: Progression-free survival
LRFS: Locoregional relapse-free survival
DMFS: Distant metastasis-free survival
CART: Classification and regression tree
AIC: Akaike information criterion
ROC: Receiver operating characteristic
AUC: Area under the ROC curves
