# Supplementary material for: Prognostic model on overall survival in elderly nasopharyngeal carcinoma patients: a recursive partitioning analysis identifying pre-treatment risk stratification
Source: Radiat Oncol. 2023 Jun 23;18:104. doi: 10.1186/s13014-023-02272-x (PMC10290415; doi:10.1186/s13014-023-02272-x)
Supplement: Supplementary file 1 — Supplementary Material 1 [file 13014_2023_2272_MOESM1_ESM.docx]

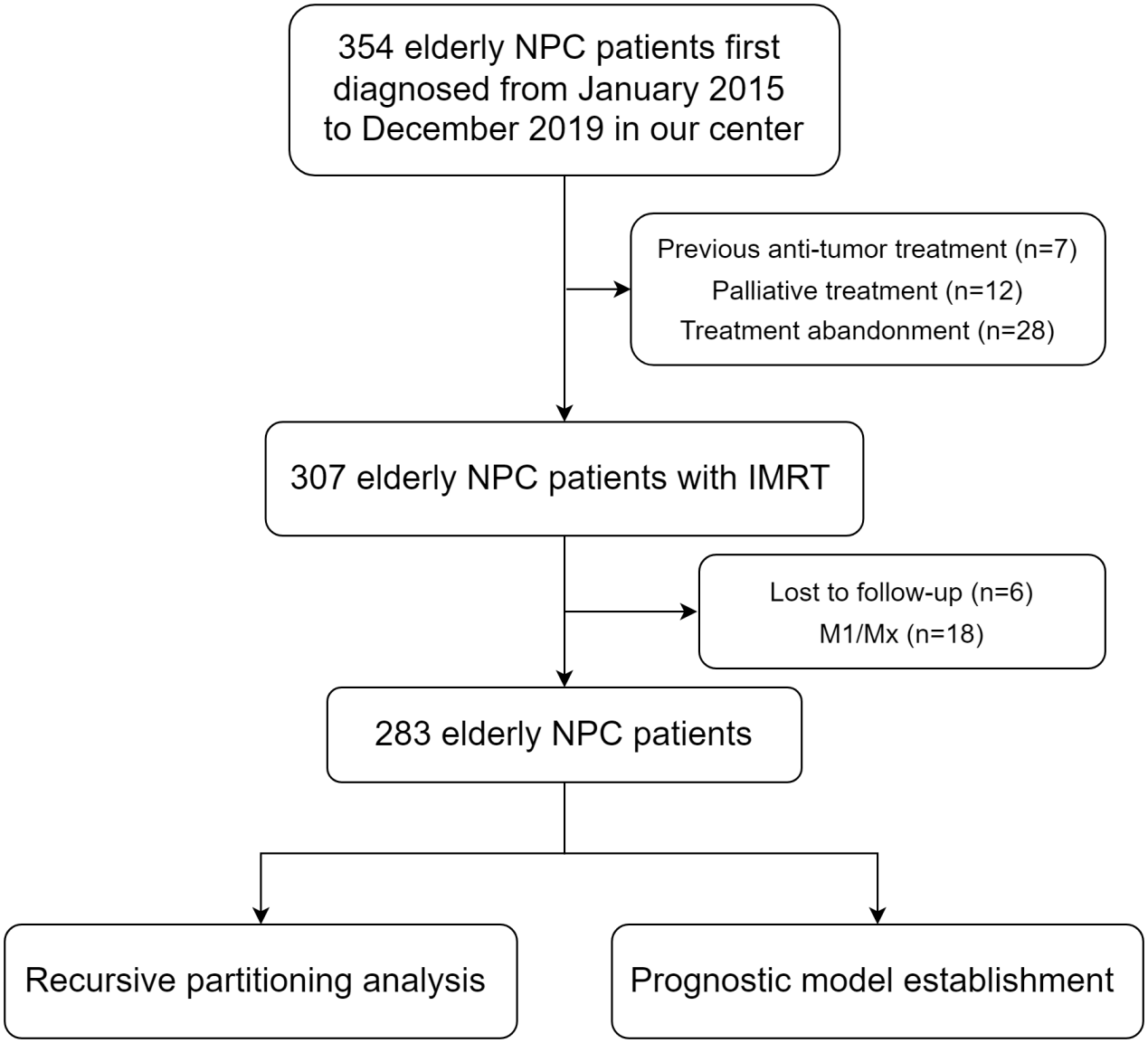


**Supplementary Fig. S1**. The flowchart of the patient inclusion and exclusion.


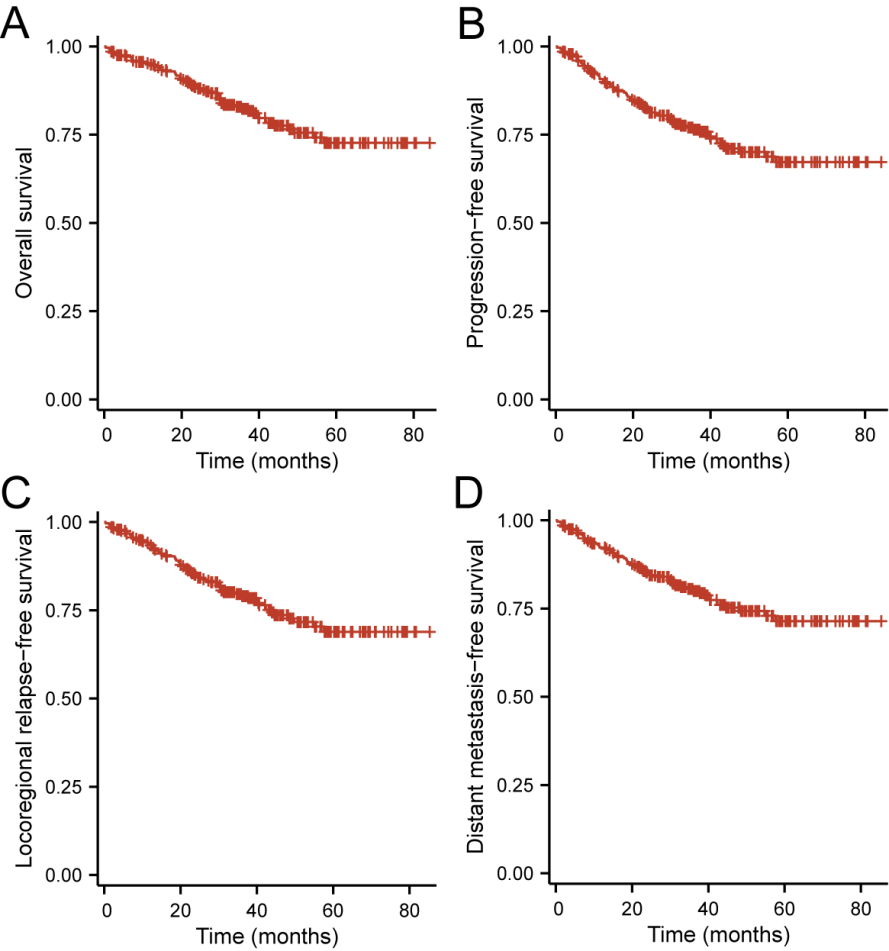


**Supplementary Fig. S2**. Kaplan-Meier survival curves of (A) overall survival, (B) progression-free survival, (C) locoregional relapse-free survival and (D) distant metastasis-free survival for the whole cohort.
